# Pre‑exposure Prophylaxis Awareness and Endorsement among Adolescents and Young Adults in Tanzania: Insights from the 2022 Demographic and Health Survey

**DOI:** 10.5334/aogh.4589

**Published:** 2025-01-24

**Authors:** Alpha Johnson Kapola, Rahma Musoke, Glenda Marie Manayon, Hassan Fredrick Fussi, Hafidha Mhando Bakari, Haji Mbwana Ally, Swalehe Mustafa Salim, Zuhura Mbwana Ally, Lynn Moshi, Mariam Salim Mbwana, Habib Omari Ramadhani

**Affiliations:** 1 EMET Healthcare, Dar es Salaam, Tanzania; 2 Water Mission, Dar es Salaam, Tanzania; 3 San Lazaro Hospital, Manila, Philippines; 4 District Hospital, Dar es Salaam, Tanzania; 5 University of Dar es Salaam, Dar es Salaam, Tanzania; 6 Kilimanjaro Christian Medical Center, Kilimanjaro, Tanzania; 7 Canada Youth Group, Dar es Salaam, Tanzania; 8 District Hospital, Tanga, Tanzania; 9 Aga Khan Hospital, Dar es Salaam, Tanzania; 10 Primary Health Care Institute, Iringa, Tanzania

**Keywords:** PrEP awareness, PrEP use endorsement, adolescents and young adults, Tanzania

## Abstract

*Background:* Pre‑exposure prophylaxis (PrEP) is an effective measure for preventing human immunodeficiency virus (HIV) infection among people at risk, including adolescents and young adults (AYA).

*Objectives:* This study evaluates the prevalence of PrEP awareness and endorsement, as well as the factors associated with these outcomes, using data from the 2022 Tanzania Demographic and Health Survey.

*Methods:* The analysis included participants aged 15–24 years. Participants were asked whether they had ever heard of daily medication to prevent HIV (PrEP) and whether they approved of daily PrEP use. Demographic characteristics and HIV testing information were also collected. Logistic regression models were used to assess factors associated with PrEP awareness and endorsement, and the weighted prevalence of these outcomes was calculated.

*Findings:* A total of 8,268 respondents aged 15–24 years were evaluated, of whom 5,852 (70.9%) were female. Overall, the prevalence of PrEP awareness and endorsement was 6.9% (95% confidence interval [CI] 6.2–7.7) and 5.7% (95% CI 5.1–6.4), respectively. Female respondents (adjusted odds ratio [aOR] = 0.69; 95% CI 0.51–0.91) and rural residents (aOR = 0.78; 95% CI 0.61–1.00) had lower odds of PrEP awareness compared with male respondents and urban residents, respectively. Previously, HIV testing and receiving test results (aOR = 2.00; 95% CI 1.50–2.66) and an age of 20–24 years versus 15–19 years (aOR = 1.54; 95% CI 1.20–1.96) were associated with increased odds of PrEP awareness. The odds of AYA endorsement of PrEP were higher among those aged 20–24 years versus 15–19 years (aOR = 1.39; 95% CI 1.07–1.83) and those who previously tested for HIV and received results (aOR = 1.97; 95% CI 1.43–2.72), but lower among female respondents (aOR = 0.66; 95% CI 0.49–0.88).

*Conclusion:* PrEP awareness and endorsement among AYA in Tanzania were low, with nearly 7 in 100 aware of PrEP and 6 in 100 approving of its use. Targeted interventions focused on adolescents, females, and rural communities are needed to increase PrEP awareness and usage to achieve the Joint United Nations Programme on HIV/Acquired Immune Deficiency Syndrome (AIDS) (UNAIDS) 95–95–95 goals for HIV epidemic control.

## Introduction

In 2022, the global human immunodeficiency virus (HIV) population was estimated at around 39 million, with 1.3 million new HIV infections reported [1]. According to the 2022–2023 Tanzania HIV Impact Survey, it was projected that approximately 1.5 million people in Tanzania would be living with HIV by 2023 [2]. The survey data also indicated an HIV prevalence of 4.5% among individuals aged 15 years or older, with approximately 60,000 new adult HIV cases identified in Tanzania during the year 2022/2023. To control the HIV epidemic, the Joint United Nations Programme on HIV/Acquired Immune Deficiency Syndrome (AIDS) (UNAIDS) set ambitious “95–95–95” targets, aiming for 95% of people living with HIV to be aware of their HIV status, 95% of those aware to be on antiretroviral treatment, and 95% of those on treatment to have suppressed viral loads. Tanzania has emerged as one of only five African countries to achieve these targets, alongside Botswana, Eswatini, Zimbabwe, and Rwanda [3].

Building on these successes in achieving the 95–95–95 UNAIDS targets, the government of Tanzania has implemented comprehensive HIV prevention measures, including the treatment‑as‑prevention strategy [4]. The government has also implemented additional prevention strategies encompassing condom programming, voluntary male circumcisions, pre‑exposure prophylaxis (PrEP), management of sexually transmitted diseases, and programs for key populations such as female sex workers and men who have sex with men, as well as vulnerable populations such as adolescent girls and young women [5]. The decline in HIV prevalence from 4.9% in 2016 to 4.5% in 2022 in Tanzania could be partially attributed to these comprehensive strategies [6, 7]. An extensive coverage of antiretrovirals from 51% in 2016 to 81% in 2022 associated with a reduction in mortality among people living with HIV could explain the minimal reduction in HIV prevalence between the two survey periods. However, the reported incidence of HIV among those who were 15 years or older was 0.24% in 2016 and 0.18% in 2022 [2, 6], equivalent to a 25% reduction in HIV incidence between the two survey periods.

Daily oral PrEP has demonstrated high efficacy in preventing HIV, with studies showing up to 99% effectiveness when taken correctly [8–10]. Despite being one of the most effective HIV prevention strategies for individuals at substantial risk of acquiring HIV, PrEP awareness and uptake remain very low among key and vulnerable populations in Tanzania. For example, among female sex workers, reported PrEP uptake and continuation rates are 55% and 47%, respectively [11, 12]. Several identified factors influence the low uptake of PrEP, including stigma, drug side effects, misconceptions, and pill burdens [11]. While oral daily PrEP faces uptake and continuation challenges, long‑acting injectable PrEP is mostly preferred and has shown improved uptake and continuation [11–13]. A recent study on lenacapavir, a long‑acting injectable PrEP that is administered twice a year, demonstrated 100% efficacy in preventing HIV among women in Uganda and South Africa [14]. These prevention outcomes underscore the importance of increasing PrEP awareness among vulnerable populations.

While research on PrEP has largely focused on key populations, such as female sex workers and men who have sex with men, limited data exist on PrEP awareness and endorsement (approval of its use) among adolescents and young adults (AYA), who are also at substantial risk of HIV acquisition. For example, in Uganda, only 23% of AYA were aware of oral daily PrEP, and awareness of injectable PrEP was as low as 3.9% (15). Understanding PrEP awareness and its endorsement among AYA is crucial for designing effective uptake strategies. Additionally, prior research has shown that, while PrEP awareness among men who have sex with men is high, actual utilization of PrEP remains low. For example, while 91% of those who were aware or who were made aware of oral daily PrEP expressed interest in using it, only 50% initiated PrEP, and merely 7.4% had detectable drug levels in their blood [15], indicating that PrEP awareness does not translate to use. Similar results have been documented among transgender AYA [16] and AYA who are members of the general student population [17]. However, data on PrEP awareness and endorsement among the broader AYA population are limited, highlighting the critical need to examine both PrEP awareness and endorsement in the general population. Therefore, this study aims to investigate the prevalence of PrEP awareness and endorsement among AYA in Tanzania using data from the 2022 Tanzania Demographic and Health Survey.

## Materials and Methods

### Survey design and data collection

The Tanzania Demographic and Health Survey (DHS) is a nationally representative, cross‑sectional household survey that was conducted in 2022. The survey used a two‑stage cluster‑based sampling design, selecting enumeration areas followed by households as previously described [2]. The survey collected information including but not limited to demographic characteristics and HIV testing. Furthermore, the survey also collected data on HIV prevention knowledge, PrEP awareness, and endorsement of PrEP use. To ensure the collection of complete data and confidentiality, the Demographic and Health Survey provided 4 weeks of training to interviewers. The Demographic and Health Survey did not collect personal identifiers and anonymized geographic information and other information that could link individuals. All interviews were conducted in private settings between the interviewer and the interviewee. Information about training and interviewing has been detailed in the Demographic and Health Survey interviewer’s training manual [18].

### Eligibility

All respondents aged 15–24 years who participated in the Tanzania Demographic and Health Survey who responded to questions about awareness of PrEP, PrEP use, and HIV knowledge were included in this analysis.

### Variables and definitions

To assess PrEP awareness, participants were asked whether they had ever heard of a medicine taken daily that can prevent a person from getting HIV. Those who had ever heard about this medicine were considered aware of PrEP. Knowledge about PrEP was reiterated, and all participants were asked whether they approved of people taking PrEP every day to prevent getting HIV. To assess knowledge of HIV prevention/transmission, participants were asked the following six questions: “Can people get HIV from mosquito bites?” “Can people reduce their chance of getting HIV by using a condom every time they have sex?” “Can people get HIV by sharing food with a person who has HIV?” “Can a healthy‑looking man have HIV?” “Can you buy vegetables from a vendor with HIV?” “Should children with HIV be allowed to attend school with children without HIV?” Questions with the correct response were assigned a score of “1,” and questions with an incorrect response were assigned a score of “0.” Scores were summed (score range: 0–6), and the Cronbach’s alpha was 0.62. For this analysis, scores were treated as continuous variables; the higher the score, the better the knowledge of HIV prevention/transmission. Age was categorized as 15–19 and 20–24 years; education as no education, primary education, or higher education; marital status as never married, married, living together, divorced/separated, or widowed; and residence as urban or rural. The wealth index was used to categorize the wealth index scores into quartiles (lowest, second, middle, and highest). Self‑reported HIV testing information was categorized as ever tested for HIV and received test results versus never tested. We hypothesized that being young, living in a rural area, having a low social economic status, having a low level of education, not testing for HIV, and having low HIV knowledge would be associated with a decreased likelihood of being aware of PrEP and approval of its use. To provide a comprehensive picture of a population’s health and demographic characteristics, the Demographic and Health Survey collects different variables, including but not limited to age, gender, education, residency, social economic status, HIV testing, and HIV knowledge over time. Among other things, these data enable researchers to analyze trends, identify health disparities, and provide recommendations on the effectiveness of health interventions.

### Statistical analysis

The weighted prevalence and 95% confidence intervals (CI) for PrEP awareness and endorsed use were computed as a proportion of the persons sampled. We compared participant covariate characteristics by the status of PrEP awareness and endorsed use. Categorical variables were compared using Chi‑squared tests and continuous variables using independent *t*‑tests. For categorical variables, we reported weighted percentages, and for continuous variables, we reported means and standard deviations. We used bivariate and multivariable logistic regression models to compute odds ratios (OR) for factors associated with PrEP awareness and endorsed use. Bivariate analysis was performed to assess the initial association of participants’ characteristics with the two outcomes. Multivariable analysis was performed to understand the final association between participants’ characteristics and the two outcomes while adjusting for potential confounders. Logistic regression models accounted for survey weights, stratification, and clustering in the sample design. We considered sociodemographic and other characteristics in bivariate analysis. All variables that were assessed in bivariate analysis were included in the final multivariable model. There were no missing data, and therefore complete case analyses were performed in the regression models. All analyses were conducted in SAS 9.4 (Cary, NC).

### Ethical considerations

These Demographic Health Surveys received approval from the Tanzania National Institute of Medical Research and the Institutional Review Board of ICF International. All adult respondents gave informed consent. Authors of this manuscript submitted a proposal to the Demographic Health Survey Program/ICF International and received permission to download and use the data for this study. The Demographic Health Survey Program authorized data access, and the data were used solely for the purpose of the current study. Demographic Health Survey data are publicly available at https://dhsprogram.com/ upon request [19].

## Results

### Characteristics of the study participants

A total of 8,268 participants were evaluated for PrEP awareness, and 8,185 were evaluated for endorsement of PrEP use. Participants’ median age was 19 years (interquartile range [IQR]: 17–22). Overall, 6.9% were aware of PrEP, and 5.7% would approve its use. Additionally, 55% of study participants were between 15 and 19 years old, 71.0% were female, and 90.1% had primary school education or higher. About two‑thirds (66.5%) were single, 65.1% were rural residents, 84.6% were on the second wealth quantile or higher, and 51.9% had ever been tested for HIV and had received test results (Table 1). The mean (standard deviation [SD]) scores for HIV knowledge were 4.6 (1.5).

**Table 1:** Characteristics of study participants.

CHARACTERISTICS	AWARENESS OF PRE‑EXPOSURE PROPHYLAXIS				ENDORSEMENT OF PRE‑EXPOSURE PROPHYLAXIS USE			
	OVERALL	AWARE	NOT AWARE	*P*‑VALUE	OVERALL	ENDORSED	NOT ENDORSED	*P*‑VALUE
	(*N* = 8,268)	(*N* = 522)	(*N* = 7,746)		(*N* = 8,185)	(*N* = 439)	(*N* = 7,746)	
**Age (years)**								
15–19	4,599 (55.3)	222 (38.8)	4,377 (56.5)	< 0.001	4,570 (54.2)	193 (41.5)	4,377 (56.5)	< 0.001
20–24	3,669 (44.7)	300 (61.2)	3,369 (43.5)		3,615 (42.9)	246 (58.5)	3,369 (43.5)	
**Gender**								
Female	5,852 (71.0)	158 (32.4)	2,258 (28.8)	0.219	2,397 (29.1)	139 (33.6)	2,258 (28.8)	0.099
Male	2,416 (29.0)	364 (67.6)	5,488 (71.2)		5,788 (70.9)	300 (66.4)	5,488 (71.2)	
**Education**								
No education	780 (9.9)	46 (8.4)	734 (10.0)	0.291	772 (9.9)	38 (8.2)	734 (10.0)	0.299
Primary education or higher	7,488 (90.1)	476 (91.6)	7,012 (90.0)		7,413 (89.1)	401 (91.8)	7,012 (90.0)	
**Marital status**								
Never married	5,655 (66.5)	316 (57.0)	5,339 (67.2)	< 0.001	5,608 (66.7)	269 (58.5)	5,339 (67.2)	0.005
Married/living together	2,294 (29.6)	177 (37.4)	2,117 (29.0)		2,263 (29.4)	146 (36.6)	2,117 (29.0)	
Divorced/separated/widowed	319 (3.9)	29 (5.6)	290 (3.8)		314 (3.9)	24 (4.8)	290 (3.8)	
**Residence**								
Urban	2,886 (34.9)	202 (41.6)	2,684 (34.4)	0.010	2,852 (34.7)	168 (39.5)	2,684 (34.4)	0.094
Rural	5,382 (65.1)	320 (58.4)	5,062 (65.6)		5,333 (65.3)	271 (60.5)	5,062 (65.6)	
**Wealth quantile**								
Lowest	1,172 (15.4)	60 (12.1)	1,112 (15.7)	0.006	1,162 (15.5)	50 (12.4)	1,112 (15.7)	0.113
Second	1,428 (17.9)	74 (13.7)	1,354 (18.2)		1,423 (18.1)	69 (15.6)	1,354 (18.2)	
Middle	1,741 (19.8)	111 (19.7)	1,630 (19.8)		1,725 (19.8)	95 (21.0)	1,630 (19.8)	
Fourth	1,841 (22.4)	116 (23.2)	1,725 (22.3)		1,815 (22.3)	90 (20.8)	1,725 (22.3)	
Highest	2,086 (24.5)	161 (31.1)	1,925 (24.0)		2,060 (24.3)	135 (30.1)	1,925 (24.0)	
**Tested for HIV and received results**								
No	4,104 (48.1)	180 (30.3)	3,924 (49.4)	< 0.001	4,078 (48.4)	154 (31.7)	3,924 (49.4)	< 0.001
Yes	4,164 (51.9)	342 (69.7)	3,822 (50.6)		4,107 (51.6)	285 (68.3)	3,822 (50.6)	
**Knowledge of HIV prevention**								
Mean (SD)	4.6 (1.5)	4.6 (1.5)	4.8 (1.3)	0.002	4.6 (1.5)	4.6 (4.6)	4.8 (1.3)	0.012

HIV means human immunodeficiency virus.

### Factors associated with PrEP awareness: bivariate analysis

Compared with those who were 15–19 years old, participants who were 20–24 years had higher odds of being aware of PrEP (OR = 2.05; 95% CI 1.65–2.54) (Table 2). Other participant characteristics that were associated with higher odds of PrEP awareness include a marital status of married/living together versus never married (OR = 1.52; 95% CI 1.20–1.93) or divorced/separated/widowed versus never married (OR = 1.71; 95% CI 1.06–2.76) and ever having been tested for HIV and having received test results compared with never having been tested (OR = 2.25; 95% CI 1.77–2.86). The odds of being aware of PrEP increase for every unit increase in the score of knowledge of HIV prevention (OR = 1.12; 95% CI 1.03–1.22). Rural residents (OR = 0.74; 95% CI 0.56–0.94) had lower odds of being aware of PrEP compared with those of residents of urban areas.

**Table 2:** Factors associated with PrEP awareness among adolescents and young adults in Tanzania, 2022.

CHARACTERISTICS	AWARENESS	BIVARIATE ANALYSIS		MULTIVARIABLE ANALYSIS	
	*N* (%)	OR (95% CI)	*P*‑VALUE	aOR (95% CI)	*P*‑VALUE
**Age (years)**					
15–19	222 (4.9)	Ref.		Ref.	
20–24	300 (9.5)	2.05 (1.65–2.54)	< 0.001	1.47 (1.13–1.90)	0.004
**Gender**					
Male	158 (7.7)	Ref.		Ref.	
Female	364 (6.6)	0.84 (0.64–1.11)	0.219	0.66 (0.50–0.88)	0.005
**Education**					
No education	46 (5.9)	Ref.		Ref.	
Primary education or higher	476 (7.1)	1.22 (0.84–1.77)	0.294	1.15 (0.78–1.70)	0.294
**Marital status**					
Never married	316 (5.9)	Ref.		Ref.	
Married/living together	177 (8.8)	1.52 (1.20–1.93)	< 0.001	1.15 (0.86–1.55)	0.344
Divorced/separated/widowed	29 (9.8)	1.71 (1.06–2.76)	0.028	1.21 (0.72–2.03)	0.467
**Residence**					
Urban	202 (8.3)	Ref.		Ref.	
Rural	320 (6.2)	0.74 (0.56–0.94)	0.013	0.72 (0.60–0.99)	0.049
**Wealth quantile**					
Lowest	60 (5.5)	Ref.		Ref.	
Second or higher	462 (7.2)	1.34 (0.98–1.83)	0.064	1.12 (0.81–1.55)	0.482
**Tested for HIV and received results**					
No	180 (4.4)	Ref.		Ref.	
Yes	342 (9.3)	2.25 (1.77–2.86)	< 0.001	1.88 (1.38–2.56)	< 0.001
**Knowledge of HIV prevention**		1.12 (1.03–1.22)	0.008	1.04 (0.95–1.14)	0.379

*N* indicates the number of people. Ref. indicates the reference category. HIV means human immune deficiency virus; OR means odds ratio; aOR means adjusted odds ratio; CI means confidence interval.

### Factors associated with PrEP awareness: multivariable analysis

Upon multivariable analysis, age, having been tested for HIV and having received test results, and place of residence continued to be associated with PrEP awareness. For example, participants who had ever been tested for HIV and had received test results had 88% higher odds of being aware of PrEP compared with those who had never been tested for HIV (OR = 1.88; 95% CI 1.38–2.56) (Table 2). Female and rural residents had 12% and 28% lower odds of being aware of PrEP compared with male and urban residents, respectively. Although married and divorced/separated individuals were associated with increased odds of PrEP awareness compared with singles on bivariate analysis, this association was lost on multivariable analysis, indicating marital status as a possible confounder for this association. Knowledge of HIV was associated with increased odds of PrEP awareness on bivariate analysis, but not on multivariable analysis. Although the questions used to assess knowledge of HIV are plausible, the calculated reliability of the questions used was 62%, indicating a satisfactory level of internal consistency [20]. The use of a validated tool with a high reliability coefficient would consistently provide a similar association upon bivariate and multivariable analyses.

### Factors associated with PrEP endorsement use: bivariate analysis

Compared with those who were 15–19 years old, participants who were 20–24 years had higher odds of endorsing the use of PrEP (OR = 1.84; 95% CI 1.45 – 2.33) (Table 3). Other participant characteristics that were associated with PrEP use endorsement include a marital status of divorced/separated/widowed versus never married (OR = 1.45; 95% CI 1.14–1.84) and ever having been tested for HIV and having received test results compared with never having been tested (OR = 2.10; 95% CI 1.61–2.73). The odds of PrEP use endorsement increase for every unit increase in the score of knowledge of HIV prevention (OR = 1.11; 95% CI 1.02–1.21).

**Table 3:** Predictors of PrEP use endorsement among adolescents and young adults in Tanzania, 2022.

CHARACTERISTICS	ENDORSEMENT OF USE	BIVARIATE ANALYSIS		MULTIVARIABLE ANALYSIS	
	*N* (%)	OR (95% CI)	*P*‑VALUE	aOR (95% CI)	*P*‑VALUE
**Age (years)**					
15–19	193 (4.3)	Ref.		Ref.	
20–24	246 (7.6)	1.84 (1.45–2.33)	< 0.001	1.34 (1.00–1.80)	0.048
**Gender**					
Male	139 (6.6)	Ref.		Ref.	
Female	300 (5.4)	0.80 (0.61–1.04)	0.099	0.64 (0.47–0.86)	0.004
**Education**					
No education	38 (4.7)	Ref.		Ref.	
Primary education or higher	401 (5.8)	1.25 (0.82–1.90)	0.301	1.18 (0.77–1.81)	0.431
**Marital status**					
Never married	269 (5.0)	Ref.		Ref.	
Married/living together	146 (7.2)	1.45 (1.14–1.84)	0.002	1.14 (0.85–1.53)	0.390
Divorced/separated/widowed	24 (7.1)	1.44 (0.85–2.44)	0.171	1.08 (0.61–1.90)	0.788
**Residence**					
Urban	168 (6.5)	Ref.		Ref.	
Rural	271 (5.3)	0.80 (0.62–1.04)	0.098	0.84 (0.64–1.11)	0.221
**Wealth quantile**					
Lowest	50 (4.6)	Ref.		Ref.	
Second or higher	389 (6.0)	1.31 (0.93–1.86)	0.125	1.13 (0.79–1.63)	0.492
**Tested for HIV and received results**					
No	154 (3.8)	Ref.		Ref.	
Yes	285 (7.6)	2.10 (1.61–2.73)	< 0.001	1.87 (1.33–2.63)	< 0.001
**Knowledge of HIV prevention**		1.11 (1.02–1.21)	0.019	1.04 (0.94–1.14)	0.456

*N* indicates the number of people. Ref. indicated reference category. HIV means human immune deficiency virus; OR means odds ratio; aOR means adjusted odds ratio; CI means confidence interval.

### Factors associated with endorsement use: multivariable analysis

Upon multivariable analysis, the association between age, having been tested for HIV and having received test results, and endorsement of PrEP use persisted. Female respondents had a 36% reduction in the odds of endorsing the use of PrEP compared with male respondents (OR = 0.64; 95% CI 0.47–0.86). As with PrEP awareness results, marital status remained a potential confounder for its association with PrEP use endorsement. Compared with singles, married individuals tended to have increased odds of PrEP use endorsement on bivariate analysis; however, this association was lost upon multivariable analysis.

## Discussion

In this study, we evaluated the prevalence of PrEP awareness and endorsement among adolescents and young adults in Tanzania using data from the 2022 nationally representative Demographic and Health Survey. Our findings reveal that PrEP awareness and endorsement are low in this population, with only 6.9% being aware of PrEP and 5.7% endorsing its use. Those who were between 15 and 19 years old, who were female, and who had never been tested for HIV were all associated with decreased odds of both PrEP awareness and approval of its use. Residents of rural areas were also associated with lower odds of PrEP awareness. These data highlight key demographic disparities in PrEP knowledge and attitudes.

To our knowledge, this is the first nationally representative analysis examining both PrEP awareness and endorsement specifically among AYA in Tanzania using recent 2022–2023 DHS data. While previous research has focused primarily on key populations or specific geographic regions, our study provides comprehensive insights into awareness and attitudes across both urban and rural settings. The identification of significant gender disparities, particularly lower awareness and endorsement among young women despite their higher HIV risk, represents a critical finding for informing targeted interventions. Additionally, the strong association between HIV testing history and both PrEP awareness and endorsement suggests an important but minimally documented opportunity for PrEP education and promotion.

The prevalence of PrEP awareness and endorsement of its use among AYA in Tanzania is low but consistent with findings reported in other countries in Africa [21, 22]. Population‑based survey data from 14 countries in Africa, including Tanzania, revealed that AYA aged 15–24 years had higher rates of recent HIV infection compared with adults aged 35–49 years old [23]. This concerning trend is evident in Tanzania’s HIV and Impact Surveys, which showed HIV incidence among adolescents and young women more than doubled from 0.14% in 2016/2017 to 0.33% in 2022/2023 [2, 6]. The heightened HIV risk among AYA can be attributed to developmental transformations and biological changes [24], compounded by behavioral factors, including low rates of condom use, high rates of sexually transmitted infections, alcoholism, and substance use [25]. Given this vulnerability and PrEP’s demonstrated effectiveness, expanding PrEP awareness and uptake among AYA at risk of HIV infection should be a public health priority.

The significant gender disparity in PrEP awareness and endorsement highlights the need for targeted, gender‑sensitive interventions for young women. Our findings that women are less likely to both be aware of PrEP and approve of PrEP use are particularly concerning, as adolescent girls and young women account for 80% of new HIV infections in Tanzania [26]. While oral daily PrEP effectively reduces HIV risk when taken as recommended, multiple barriers impede PrEP uptake, including migration, food insecurity, perceived low risk, loss of interest, side effects, and stigma [27]. The emergence of long‑acting injectable PrEP offers a promising alternative, given its demonstrated acceptability and effectiveness. To maximize impact and achieve meaningful public health benefits, PrEP services should be integrated into the existing reproductive health infrastructure and services, with particular attention to addressing barriers in demand creation, adherence support, and service delivery models [28].

Addressing the urban–rural divide in PrEP awareness and uptake requires scalable interventions that effectively leverage existing healthcare resources in rural areas. Potential strategies include community‑based outreach programs, mobile health units, and the use of technology‑enabled communication channels (e.g., radio, mobile messaging, social media) to disseminate PrEP information to rural populations. The disparity in PrEP awareness between rural and urban areas is not unique to Tanzania (and has been reported elsewhere) [29]; studies have shown that rural healthcare providers are less comfortable than their urban counterparts with delivering and providing HIV‑risk‑reduction counseling to adolescents and communicating about their positive HIV test results [30]. To bridge this gap, leveraging skills from urban providers and establishing urban–rural collaborative partnerships that facilitate knowledge transfer and skill building among healthcare providers could strengthen PrEP awareness initiatives in rural areas. Additionally, HIV testing campaigns present valuable opportunities to educate AYA about PrEP, particularly reaching those who may not otherwise access traditional healthcare services.

The strong association between HIV testing and both PrEP awareness and endorsement suggests that HIV testing services could serve as a crucial intervention point for increasing PrEP knowledge and acceptance among AYA. Integration of PrEP education and promotion into existing HIV testing services could provide a practical and cost‑effective strategy for reaching this population. This approach could be particularly effective when combined with targeted interventions for specific subgroups—such as school‑based programs for younger adolescents, integrated reproductive health services for young women, and mobile health units for rural communities. Future research should evaluate the effectiveness of these integrated approaches and explore innovative strategies for addressing barriers to PrEP uptake and adherence among AYA.

We recognize both the strengths and limitations of this study. The main strength lies in the use of nationally representative data, which enables robust inferences about PrEP awareness and endorsement among people aged 15–24 years old in Tanzania. The large sample size and rigorous data collection methodology, incorporating careful stratification and weighting, enhance the validity and generalizability of our findings. Our comprehensive analysis of detailed sociodemographic factors, including age, gender, education, marital status, and urban–rural residence, provides nuanced insights into PrEP awareness and endorsement patterns across diverse populations and geographic regions, informing targeted public health interventions.

However, this study has important limitations. The cross‑sectional design of the study prevents us from establishing causal inferences about the relationships between the examined factors and PrEP awareness or endorsement. Additionally, while the DHS data provide broad population coverage, they may not capture detailed information about specific barriers to PrEP awareness and endorsement that could be better understood through qualitative research.

## Conclusion

In conclusion, our findings suggest that PrEP awareness and endorsement are low among adolescents and young adults in Tanzania, with significant disparities based on gender, HIV testing history, and rural residence. To achieve the UNAIDS 95–95–95 goals for HIV epidemic control, targeted interventions focused on increasing PrEP awareness and uptake among adolescents, females, and rural communities are urgently needed. Addressing barriers such as stigma, healthcare access, and adherence to daily oral PrEP is essential for reducing HIV incidence in this vulnerable population.

### Data availability statement

Third‑party data were obtained for this study from the DHS Program (https://dhsprogram.com/). Data may be requested from the DHS Program after creating an account and submitting a concept note. More access information can be found on the DHS Program website (https://dhsprogram.com/data/Access-Instructions.cfm). The data set is openly available upon permission from the MEASURE DHS website (https://www.dhsprogram.com/data/available-datasets.cfm). The authors confirm that interested researchers would be able to access these data in the same manner as the authors. The authors also confirm that they had no special access privileges that others would not have.
